# Notes of a Case of Imitative Neuralgic Affection, Occasioned by Mercury

**Published:** 1829-03

**Authors:** J. Bennett

**Affiliations:** Newport, Ky.


Art. III.—*Notes of a case imitative of Neuralgic affection, occasioned by Mercury.* By Doctor J. Bennett.

In the Spring 1825, I was consulted by a man who had contracted syphilis; and found him labouring under chancre, bubo, and an eruption on the skin. I put him on a course of mercury, and directed confinement to his room. The mercury soon produced a ptyalism; and from its first affecting the mouth, he complained of unusual pain in the right cheek, which I took but little notice of, supposing it arose from decayed teeth. As the soreness of the mouth increased, the painful affection of the cheek became almost insupportable. On attempting to speak, the muscles of the right cheek were thrown into violent spasms; indeed, every motion of the face was followed by a paroxysm: the pain, following the course of some of the principal nerves of the face, indicated its affinity to tic douloureux.

To remedy this affection, I had recourse to opium, both internally and externally, using a watery extract of solution, as a wash to the mouth. Although this medicine was not sparingly exhibited, it increased the painful affection of the face to an intolerable degree, and the spasmodic twitchings became almost incessant. On the day following the exhibition of the opium, I directed him to take Bark, hourly, in as much wine as would form a convenient vehicle. On visiting him in the evening, I found him free from pain; he informed me that he had attempted to take the bark; that it had vomited him and produced violent pain and spasms, in the muscles of the face. For relief my patient flew to the wine bottle, half a pint of which he drank at a single draught; which tranquilized the spasms and suspended the pain in a few minutes. He subsequently drank the same quantity through the day, resorting to it whenever he felt the pain returning, with immediate relief. His mouth was less sore this evening, and the discharge of saliva had evidently increased.

Wishing again to try the effects of opium, I directed him to discontinue the wine during the night, and to take that medicine in combination with camphor. In the morning, I found him suffering the most excruciating pain, which came on soon after he had taken the opium, and continued to torment him through the night. The wine was now resumed and again afforded immediate relief. From this time the patient suffered little or no pain. He kept up a moderate excitement with the wine until his mouth was well, which happened sooner than any case of sore mouth from mercury, which I recollect to have seen. A subsequent salivation in the same patient produced a similar disease of the muscles of the face, and the same remedies produced the same effects as before. I ought to remark that the patients teeth were sound.

Is mercurial sore mouth more speedily cured by a liberal use of wine, or ardent spirits, than by the ordinary remedies? Does not the free use of wine produce a discharge of saliva in mercurial cases, attended with dryness and ulceration of the mouth, with hemorrhages from the gums? May we not infer from this case, that wine would give relief in certain forms, of true tic douloureux?

*Newport, Ky. January* 18, 1829.

